# A Pilot Study of the Association of Tumor Necrosis Factor Alpha Polymorphisms with Psoriatic Arthritis in the Romanian Population

**DOI:** 10.3390/ijms12085052

**Published:** 2011-08-08

**Authors:** Olivia M. Popa, Mihai Bojinca, Violeta Bojinca, Monica I. Dutescu, Mihaela Meirosu, Ruxandra E. Caisan, Claudia Ciofu, Constantin Bara, Luis O. Popa

**Affiliations:** 1 Department of Immunology and Pathophysiology, Faculty of Medicine, University Carol Davila, Bucharest 041914, Romania; E-Mails: oliviapopa@yahoo.com (O.M.P); caravi.bara@gmail.com (C.B.); 2 Department of Rheumatology, Faculty of Medicine, University Carol Davila, I.C. Cantacuzino Hospital, Bucharest 020475, Romania; E-Mails: mihaibojinca@yahoo.com (M.B.); claudia.ciofu@gmail.com (C.C.); 3 Department of Rheumatology, Faculty of Medicine, University Carol Davila, St. Maria Hospital, Bucharest 011172, Romania; E-Mail: vmbojinca@yahoo.com; 4 “Prof. Dr. C. T. Nicolau” National Institute of Blood Transfusion, Bucharest 011155, Romania; E-Mails: monica_dutescu@yahoo.com (M.I.D.); ru.caisan@gmail.com (R.E.C.); 5 ICA Research and Development, Bucharest 013721, Romania; E-Mail: mihaelameirosu@yahoo.com; 6 Molecular Biology Department, Grigore Antipa National Museum of Natural History, Sos. Kiseleff Nr. 1, Bucharest 011341, Romania

**Keywords:** tumor necrosis factor alpha (TNF-alpha), psoriatic arthritis, single nucleotide polymorphism (SNP), undifferentiated spondyloarthritis

## Abstract

Tumor necrosis factor alpha (TNF-alpha) is an important pro-inflammatory cytokine implicated in the pathogenesis of psoriatic arthritis. We have performed a case-control association study of three TNF-alpha gene polymorphisms in a group of Romanian psoriatic arthritis patients *versus* ethnically matched controls. A second group of patients with undifferentiated spondyloarthritis was used in order to look for similarities in the genetic background of the two rheumatic disorders. The −857C/T polymorphism was associated with susceptibility to psoriatic arthritis in our population at the individual level (*p* = 0.03, OR 1.65, 95% CI 1.05–2.57) and in combined haplotypes with the −238G/A and −308G/A SNPs. Regarding the investigated polymorphisms and derived haplotypes, no potential association was found with the susceptibility to undifferentiated spondyloarthritis in Romanian patients.

## 1. Introduction

The regulatory proteins known as cytokines have an important role in the pathogenesis of inflammatory diseases [1,2]. Among them, tumor necrosis factor-alpha (TNF-alpha) was intensively studied in relation with these disorders. Although anti-TNF-alpha therapies represent a major step in the treatment of many inflammatory conditions [3–5], the pathological involvement of TNF-alpha is not completely understood.

Psoriatic arthritis (PsA) is a rheumatic disease that belongs to the group of spondyloarthritides. The disease is characterized by inflammatory arthritis, localized at the axial skeleton and/or peripheral joints, which is accompanied in most cases by psoriatic skin lesions [6]. Given the pattern of inheritance, PsA is considered a genetically complex multigenic disease [7]. Single nucleotide polymorphisms (SNPs) of the TNF-alpha gene showed association with psoriatic arthritis in some populations [8,9], but no association in others [10,11]. TNF-alpha is located in the major histocompatibility complex (MHC) region on the chromosome 6p21.3, region that showed the strongest signal of association in the genome-wide association study that included psoriatic arthritis patients [12].

Undifferentiated spondyloarthritis (USpA) is the second most common subtype of seronegative spondyloarthritides, but it is less studied from the point of view of genetic association compared with other diseases in this group, like ankylosing spondylitis and psoriatic arthritis [13,14]. An association of TNF-alpha -308G/A polymorphism with susceptibility to USpA was found in a group of Mexican patients [15].

Our aim in this study was to investigate the association of three TNF-alpha gene promoter polymorphisms with psoriatic arthritis in a Romanian group of patients. A second cohort of undifferentiated spondyloarthritis patients was included in the study in order to find similarities in the genetic background of the two inflammatory diseases. This is the first study of TNF-alpha SNPs in a Romanian group of spondyloarthritides.

## 2. Results and Discussion

We have performed a case-control association study of TNF-alpha gene single nucleotide polymorphisms in a group of Romanian psoriatic arthritis patients *versus* ethnically matched controls. A second group of patients with undifferentiated spondyloarthritis was used in order to look for similarities in the genetic background of the two rheumatic disorders.

None of the three TNF-alpha polymorphisms showed a departure from Hardy-Weinberg equilibrium in the investigated groups. The data for controls were compared with data available in the database www.allelefrequencies.net for Caucasian populations. The genotype frequencies of the −308G/A and −238G/A polymorphisms were in the range of those reported for other populations. The rare genotype −238AA was not present in the investigated Romanian control group or patients. For the −857C/T polymorphism, only two Caucasian populations have been reported in the database, with frequencies of CC genotype significantly higher than ours (82% *versus* 67%, *p* = 0.004, for a population from Norway, respectively 88% *versus* 67%, *p* = 0.0001, for a Canadian population).

The analysis of alleles and genotypes distribution of each of the individual SNPs in cases compared with controls revealed a positive uncorrected association of the −857C/T polymorphism with psoriatic arthritis, but not with undifferentiated spondyloarthritis (Table 1). The minor allele T was more prevalent in PsA patients than in controls (*p* = 0.03) and the genotypes bearing T allele were significantly more frequent in PsA patients (*p* = 0.01). The distribution of the TNF-alpha −238G/A and −308G/A polymorphisms was not significantly different between patients and controls in both disease groups.

The analysis of the three subgroups of PsA patients revealed similar distribution of the investigated polymorphisms in oligoarthritis and polyarthritis patients and a high frequency of −857T allele in the subgroup of spondylitis patients (30.3% *versus* 19% in controls), but this result did not reach a statistical significance (*p* = 0.07).

The correlation coefficient between each pair of SNPs (r2) was below 0.1 (Table 2), so all the three investigated SNPs were included in the haplotype analysis. Four haplotype combinations between the three polymorphic loci of TNF-alpha were observed in each group (Table 3). The haplotype −857C/−308G/−238A exhibited a frequency of only 2% in controls and was eliminated from further analysis. The frequency of −857T/−308G/−238G haplotype tends to increase significantly in PsA patients with respect to healthy controls (*p* = 0.02). The analysis of the estimated haplotypes between pairs of SNPs (Table 3) revealed a positive association of those combinations that contains the minor −857T allele (−857T/−308G, *p* = 0.02 and −857T/−238G, *p* = 0.02) and a negative association of −857C/−238G haplotype (67% in cases *versus* 78% in controls, *p* = 0.006). A negative association of this haplotype (−857C/−238G) was found also in the PsA patients with spondylitis (64% in cases *versus* 78% in controls, *p* = 0.01). No association of any of the estimated haplotypes was found in the USpA group.

Tumor necrosis factor alpha is an important pro-inflammatory cytokine that is considered to play an essential role in driving the cytokine cascade at sites of inflammation both of the skin and joints of PsA patients [16]. High levels of TNF-alpha found in the inflammatory infiltrate of the synovium and in membranes of the affected joints [17–19] made TNF-alpha an attractive candidate for genetic research and for biological therapies. PsA patients showed decreased serum levels of TNF-alpha during efficacious treatment with TNF blocking agents [16]. A possible mechanism of TNF-alpha pathological contribution to the development of PsA could be through interleukine 23 (IL-23) that has a leading role in the development of cells producing TNF-alpha. SNPs of the gene coding for p40 subunit of IL-23 show association with both psoriatic arthritis and psoriasis in recent studies [20].

Studies on TNF-alpha gene polymorphisms in relation to psoriatic arthritis generated conflicting results in different populations. No association was found in Jewish and Spanish populations [10,21]. We have found that TNF-alpha −857C/T polymorphism, previously reported as being associated with PsA in a German group [22], also represents a risk factor for this condition in the Romanian population. However, this result looses statistical significance if the correction for multiple testing is applied. Significant associations were found between −238G/A polymorphism and PsA in the German and Canadian populations [8,9], an association that was not confirmed in the present study. Moreover, the relation of strong linkage disequilibrium found between the three TNF-alpha SNPs in a German group [22] was not present in the Romanian cohort. These variations from one study to another might reflect the differences in the genetic background of the populations, but also the variability of the disease itself. In our PsA group, the spondylitis and polyarthritis patterns were about 37% each, the rest being oligoarthritis. In the German PsA group, only 19.2% of cases had spondylitis and about 69% had polyarthritis. In the Canadian study, 61% of patients presented polyarthritis, 5.2% spondylitis and the rest oligoarthritis and other arthritis patterns.

We have identified several haplotypes associated with the susceptibility of this disease and also a haplotype associated with a specific pattern of arthritis (spondylitis). To our knowledge, this association has not been previously found in any population. Future studies are needed to elucidate the precise contribution of this genomic region to the pathogenesis of psoriatic arthritis.

Regarding undifferentiated spondyloarthritis, the investigated TNF-alpha polymorphisms did not show any association in this Romanian group of patients. The inflammatory arthritis of this disorder seems to have different genetic causes compared with psoriatic arthritis in our population. The association found in the Mexican population^15^ could be the result of a different ethnic background.

## 3. Experimental Section

### 3.1. Subjects

Two groups consisting of 86 unrelated Romanian patients with psoriatic arthritis (M/F 42/44, mean age 51 years) and 79 Romanian patients with undifferentiated spondyloarthritis (M/F 30/49, mean age 37.5 years) were recruited from two rheumatology centers (“St. Maria” Hospital and “I.C. Cantacuzino” Hospital, Bucharest, Romania) between 2007 and 2010. The PsA patients satisfied the Classification criteria for the diagnosis of Psoriatic Arthritis (CASPAR) [23]. Among the psoriatic arthritis patients, 22 subjects (26%) had peripheral arthritis with less than 5 joints affected (oligoarthritis), 32 individuals (37%) had more than five joints affected (polyarthritis) and 32 subjects (37%) presented arthritis with axial involvement (spondylitis). All undifferentiated spondyloarthritis patients fulfill the European Spondyloarthropathy Study Group diagnostic criteria for spondyloarthritides [24], but not the specific criteria for any other disease in this group.

A cohort of healthy Romanian individuals from “Prof. Dr. C. T. Nicolau” National Institute of Blood Transfusion, Bucharest, Romania (potential organ donors, *n* = 147) was used as control group. The controls matched the patient groups for sex ratio and mean age (1.5 and 39 years, respectively). None of the control subjects had a history of a diagnosed rheumatic condition or psoriasis.

The study was approved by the local ethics committee. The details were explained to all patients and controls and consent for genetic screening was obtained.

### 3.2. Genotyping of TNF-Alpha Promoter Polymorphisms

The genomic DNA was extracted from venous blood with the Qiagen Blood Mini Kit (Qiagen, Hilden, Germany) according to the manufacturer protocol.

Patients and controls were genotyped for three TNF-alpha gene promoter polymorphisms: −857C/T (rs 1799724), −308G/A (rs 1800629) and −238G/A (rs 361525). The genotyping of all three polymorphisms was performed by allelic discriminating TaqMan Real-Time PCR with TaqMan SNP Genotyping Assays according to manufacturer protocol (Applied Biosystems, USA) on a 7300 Real Time PCR System (Applied Biosystems, USA).

### 3.3. Statistical Analysis

Alleles and genotypes frequencies of all SNPs were obtained by direct counting. The Hardy-Weinberg equilibrium was tested using the Chi-square test. In order to evaluate the possibility of the haplotypes’ reconstruction between the investigated SNPs, the level of linkage disequilibrium (LD) was studied by calculating the correlation coefficient between each pair of loci (r2). The association test for the disease trait was performed using the Fisher’s exact test and also with a logistic regression model. The association tests for each polymorphism and haplotype, the LD and HWE tests and the haplotype frequency estimations were performed with the software package PLINK v 1.07 [25] and *p* values ≤0.05 were considered statistically significant.

## 4. Conclusions

The present study shows that TNF-alpha gene promoter polymorphism −857C/T is associated with the susceptibility to psoriatic arthritis in Romanian population at the individual level and in combined haplotypes with −238G/A and −308G/A SNPs. These findings require further validation on a larger group of patients. No potential association was found regarding the three investigated polymorphisms and derived haplotypes with susceptibility to undifferentiated spondyloarthritis in Romanian patients.

## Figures and Tables

**Table 1.** Genotype distribution and allele frequencies of the tumor necrosis factor (TNF)-alpha gene promoter polymorphisms for psoriatic arthritis (*n* = 86), undifferentiated spondyloarthritis (*n* = 79) and controls (*n* = 147). OR, odd ratio; CI, 95% confidence interval for OR.

**SNP**	**Group**		**Genotype**		**Minor Allele Frequency**	***p* Value**	**OR/CI (95%)**
TNF-857 *		CC	CT	TT	T		
rs 1799724	PsA	46 (53.5%)	32 (37.2%)	8 (9.3%)	27.9%	**0.03**	1.65 (1.05–2.57)
	Controls	95 (67%)	41 (28.8%)	6 (4.2%)	19%		
	USpA	47 (59.7%)	27 (34%)	5 (6.3%)	23.4%	0.27	1.3 (0.81–2.08)
TNF-308 *		GG	GA	AA	A		
rs 1800629	PsA	71 (82.6%)	13 (15.1%)	2 (2.3%)	10%	0.24	0.66 (0.36–1.22)
	Controls	107 (75.4%)	30 (21.1%)	5 (3.5%)	14%		
	USpA	62 (78.6%)	16 (20.2%)	1 (1.2%)	11.4%	0.46	0.78 (0.43–1.42)
TNF-238		GG	GA	AA	A		
rs 361525	PsA	78 (90.7%)	8 (9.3%)	0	4.6%	0.15	2.34 (0.8–6.56)
	Controls	141 (96%)	6 (4%)	0	2%		
	USpA	73 (92.4%)	6 (7.6%)	0	7.6%	0.35	1.89 (0.6–5.97)

* Data available for 142 control subjects.

**Table 2.** Results of linkage disequilibrium statistics for TNF-alpha polymorphisms for Romanian psoriatic arthritis (PsA) patients and controls.

	**TNF-238G/A**	**TNF-308G/A**	**TNF-857C/T**
TNF-238G/A	1	0.00836571	0.0151408
TNF-308G/A		1	0.0740285
TNF-857C/T			1

**Table 3.** TNF-alpha haplotypes in PsA patients and controls.

**Haplotype**	**Haplotype**	**Frequency**	***p* Value**
857/308/238	PsA	Controls	
CGA	0.04	0.02	0.12
CAG	0.09	0.14	0.18
TGG	0.27	0.19	**0.02**
CGG	0.57	0.64	0.12

857/308			
CA	0.09	0.14	0.18
TG	0.27	0.19	**0.02**
CG	0.62	0.66	0.3

857/238			
CA	0.04	0.02	0.12
TG	0.27	0.19	**0.02**
CG	0.67	0.78	**0.006**
